# Alterations in Gut Microbiota Profiles of Mice Infected with *Echinococcus granulosus* sensu lato Microbiota Profiles of Mice Infected with *E. granulosus* s.l.

**DOI:** 10.1007/s11686-022-00613-6

**Published:** 2022-09-01

**Authors:** Mingxing Zhu, Chan Wang, Songhao Yang, Xiancai Du, Yazhou Zhu, Tingrui Zhang, Yongxue Lv, Wei Zhao

**Affiliations:** 1 Center of Scientific Technology of Ningxia Medical University, Yinchuan, Ningxia Hui Autonomous Region 750004 People’s Republic of China; 2 Key Laboratory of Prevention and Control of Common Infectious Diseases of Ningxia , Yinchuan, Ningxia Hui Autonomous Region 750004 People’s Republic of China; 3 Department of Medical Genetics and Cell Biology, School of Basic Medical Science of Ningxia Medical University, Yinchuan, Ningxia Hui Autonomous Region 750004 People’s Republic of China

**Keywords:** *Echinococcus granulosus* sensu lato, Cystic echinococcosis, Gut microbiota, 16S rRNA metagenome sequencing, KEGG pathways, Mice

## Abstract

**Objective:**

Cystic echinococcosis is a kind of parasitic disease that seriously endangers human and animal health. At present, its prevention and treatment still do not achieve the desired results. The aims of this study were to explore the effect of CE on intestinal microflora in mice.

**Methods:**

In this study, 16S rRNA metagenome sequencing and bioinformatics were used to analyze the intestinal flora of mice infected with *E. granulosus* s.l. Changes in intestinal microbial community abundance were investigated and the differences in microbial populations of mice infected with *E. granulosus* s.l. were screened.

**Results:**

Our results show that at the phylum level, nine abundant taxa were identified, the relative abundance of Firmicutes and Proteobacteria were enriched in infected mice, whereas Bacteroidetes and Patescibacteria were enriched in control mice (*P* < 0.01). At the class level, 13 abundant taxa were identified, the relative abundance of Bacilli was enriched in control mice, but decreased in infected mice (*P* < 0.01). At the order level, 15 abundant taxa were identified, the relative abundance of Lactobacillales was enriched in control mice, but decreased in infected mice (*P* < 0.01). At the family level, 28 abundant taxa were identified, enriched bacteria in the infected mice was Streptococcaceae, while the enriched bacteria in the control group was Lactobacillaceae (*P* < 0.01). At the genus level, 79 abundant taxa were identified, enriched bacteria in the infected mice was Streptococcus, while the enriched bacteria in the control group was uncultured_bacterium_f_Eggerthellaceae (*P* < 0.01). At the species level, 80 abundant taxa were identified, enriched bacteria in the infected mice was uncultured_bacterium_g_Streptococcus, while the enriched bacteria in the control group was uncultured_bacterium_f_Eggerthellaceae (*P* < 0.01). 39 KEGG pathways were identified that were differentially enriched between the infected and control mice.

**Conclusion:**

This study comprehensively demonstrates the differential intestinal microbiota of infected mice and analyzes the metabolic pathways related to the specific microbiota. This could provide new targets and research direction for the treatment and prevention of diseases caused by *E. granulosus* s.l.

## Introduction

Cystic echinococcosis (CE) is a parasitic disease caused by *Echinococcus granulosus* sensu lato (*E. granulosus* s.l.) that mainly occurs in countries and regions with developed animal husbandry [1, 2]. The infection rate is closely related to living habits and conditions, climate, environmental conditions, and livestock quarantine levels [3]. Infection occurs in humans, as well as in animals, such as sheep, pigs, goats, camels, buffaloes, and horses [4]. These natural intermediate hosts also have a high incidence rate in areas with high incidence of human echinococcosis. Humans and herbivores (cattle, sheep, horses, camels, etc.) are intermediate hosts. Carnivores (dogs and wolves) are the final hosts. The main causes of intermediate host infection are directly contact with dogs or ingestion of contaminated drinking water and food. The most common domestic circulation of *E. granulosus* s.l. is in dogs and sheep [5, 6]. Many studies have confirmed that the gut microbiome transmits signals to distal organs through metabolites, linking gut microbes to other organs of the host [7]. The composition and metabolism of intestinal microbiota may be an important factor in the pathogenesis of many diseases; however, there is limited information about the composition of gut microbiota and its metabolism in *E. granulosus* s.l. -infected hosts.

The contribution of intestinal microorganisms to diseases can be divided into two categories: (1) microorganisms affect the stability of the host genome, leading to host DNA mutations that promote the occurrence of diseases; (2) microorganisms induce inflammatory responses and participate in the progress of the disease in the host [8, 9]. The development and progress of a variety of human diseases are affected by the presence of specific pathogens and the metabolic output of the entire microbiota [10, 11]; for example, microbial metabolism plays an important role in the progress of a variety of cancers [14, 15]. Studies have shown that many different diseases can alter the gut microbiota, and the gut microbiota can also regulate the pathogenesis of infectious diseases [12, 13]. In one study, the difference between patients with echinococcosis and healthy individuals was reported in four phyla: Firmicutes, Proteobacteria, Bacteroidetes, and Actinobacteria. This study revealed that the changes of intestinal flora in patients with echinococcosis may be related to the development of echinococcosis [16]. Gut microbiota affect host metabolism through a variety of direct and indirect biological mechanisms, and host metabolic disorders usually lead to diseases.

There are currently no studies exploring the relationship between *E. granulosus* s.l. infection and host gut microbiota. However, the continuous development of metabonomics and microbial intestinal microflora sequencing technology, and the increased interest in disease research, suggests the relationship between parasitic diseases and gut microbiota can be investigated further.

In this study, fecal samples from *E. granulosus* s.l. infected and control mice were used for 16S rRNA gene sequencing to characterize the intestinal microbial community. Differences in microorganisms between the infected and control groups were analyzed to determine how *E. granulosus* s.l. infection affects the function and metabolism of mice through the intestinal flora. Among the different gut microbiota, some may be potential markers for the diagnosis of CE. Therefore, the gut microbiota of infected mice may provide a new non-invasive early target for disease monitoring and adjuvant therapy of CE.

## Materials and Methods

The study protocol was approved by the Institutional Review Board of Ningxia Medical University (Approval Number 2020-603).

### Sample Collection

BALB/c mice were purchased from the experimental animal center of Ningxia Medical University (Yinchuan, Ningxia Hui Autonomous Region, PR China). Eight-week-old female BALB/c mice (*n* = 20; 18–22 g) were randomly divided into two groups, namely control (*n* = 10) and *E. granulosus* infection (*n* = 10). Protoscoleces of *E. granulosus* s.l. were obtained by surgical removal of cysts from patients with CE at the General Hospital of Ningxia Medical University, Department of Hepatobiliary Surgery. Mice in the infection group were intraperitoneally injected with 2000 protoscoleces (100 μl) diluted with phosphate-buffered saline (PBS). In the control group, 100 μl PBS was injected intraperitoneally. After 6 months of observation, cysts were observable in mice in the treatment group. Multiple cysts were formed in the abdominal cavity of the mice, the largest cyst being approximately 1 cm in diameter. Fecal samples were collected for follow-up experiments.

During the experiment, all operations are carried out according to the Guide for Laboratory Animal Care and Use. At the end of the experiment, the mice were handed over to professionals to be euthanized.

### Genomic DNA Extraction and 16S rRNA Gene Sequencing

DNA was extracted from fecal samples of infected and control mice using the Tiangen DNA Extraction Kit (Tiangen biotechnology), following the manufacturer’s instructions. SDS (30 μL; 10%), proteinase K (3 μL; 20 g/L), and RNASeA (4 μL) were added to the pretreated 500 μL sample and mixed. The samples were kept in a water bath at 37 °C for 1 h. NaCl (100 µl; 5 mol/L) was added to each tube and mixed by inverting, then CTAB/NaCl (80 μl; 10% CTAB, 0.7 mol/L NaCl) was added, gently mixed, and put in a water bath at 65 °C for 10 min. An equal volume of phenol/chloroform/isoamyl alcohol (25:24:1) mixture was added, mixed, and then centrifuged for 10 min at 12,000 r/min, and the supernatant extracted. Isopropyl alcohol (0.6 vol.) was added to the supernatant, mixed gently, centrifuged at 12000r/min for 10 min, and then the supernatant was discarded. The precipitation was washed with 1 mL precooled 75% ethanol, centrifuged at 7500 r/min for 5 min, then the ethanol was discarded, slightly dried on a clean bench, and dissolved in TE buffer (30 μL). Ten fecal samples were analyzed for microbial profiling from each group.

PCR amplification and target labeling were performed with specific primers for the DNA obtained after splitting the samples. PCR products were purified, and index tags were established for target sites for the library: library purification, library quality inspection, and computer sequencing were performed after passing the quality inspection.

### Statistical Analyses

All data analyses were conducted using SPSS version 22.0. Operational Taxonomic Units (OTUs) were used to evaluate the amount of sequencing data [17]. Alpha and beta indices were used to analyze microbial diversity [18]. Wilcoxon rank-sum test and linear discriminant analysis were used to analyze the differences in abundance. The KEGG pathway was used to annotate the function of different metabolites [19].

## Results

### Microbial Profiling of Infected and Control Mice

**Fig. 1:**
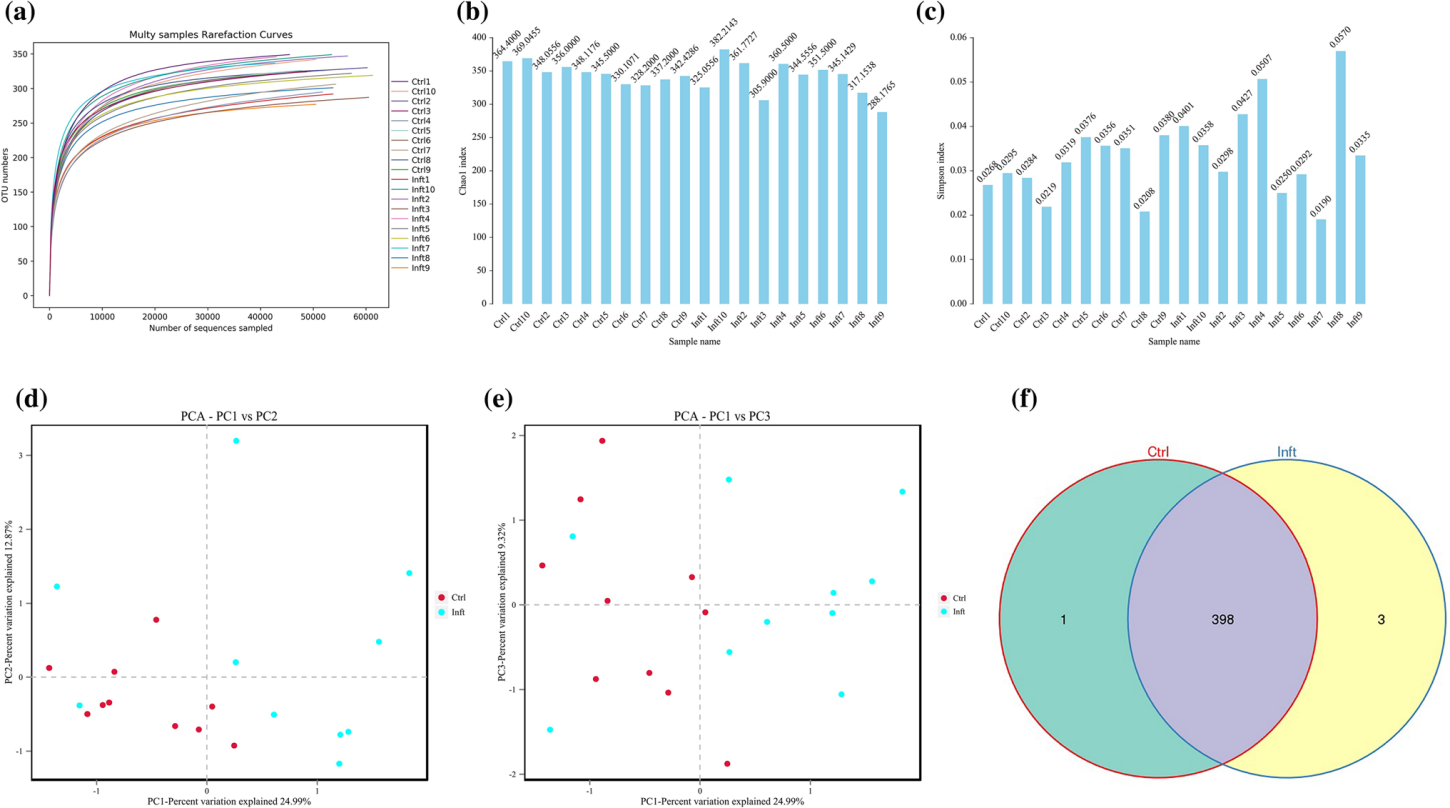
The diversity and variation of the gut microbiota in *E. granulosus* s.l. infected and control mice. **a** Rarefaction curves for gene numbers in the control (*n* = 10) and infected (*n* = 10) groups. The curve of each group was nearly smooth, indicating that the depth and quantity of sequencing data were sufficient; **b**, **c** Chao1 and Simpson indices were used to estimate the diversity of the gut microbiota; **d**, **e** principal component analysis (PCA) of Bray–Curtis analysis showed that there were significant differences between the infected and control mice; **f** Venn diagram illustrating the distribution of intestinal microorganisms (*n* = 10 per group) that may represent the overall status (infected vs. control mice) in the number and category of operational taxonomic unit (OTUs). (OTU: operational taxonomic unit; Ctrl: control; Inft: infected)

From the 20 fecal samples, 6422 OTUs were used to analyze gut microbiota richness, diversity, and composition. The sequencing depth was detected by drawing the sparse curve of richness; the curves of each group were close to saturation, indicating that the sequencing depth was sufficient (Fig. 1a). The Wilcoxon rank-sum test depicted the species richness (Chao1 index) (Fig. 1b), while the Simpson index measured both richness and uniformity in the infected and control mice (Fig. 1c). To assess the degree of similarity between microbial communities, the beta-diversity was calculated by the Bray–Curtis method, and a principal component analysis (PCA) was performed. There were significant differences in microbial communities between infected mice and control mice (Fig. 1d and e). A Venn diagram was used to identify the gut microbial profile that represented the overall status. A comparison of microbial profiles of infected and control mice revealed that there were 402 common OTUs and four commonly altered genera (Fig. 1f).

### Gut Microbiota in Infected and Control Mice

**Fig. 2:**
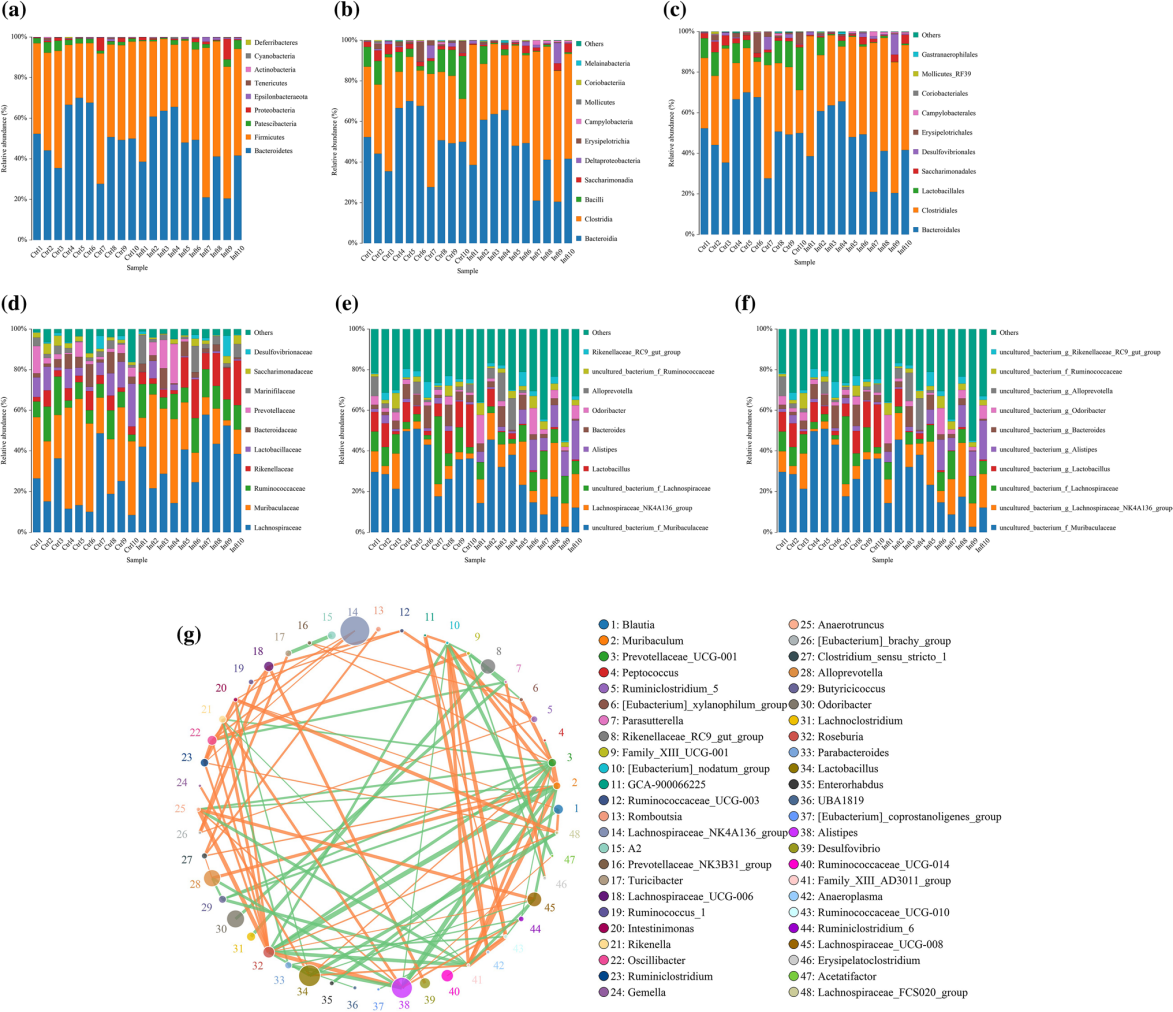
Changes of gut microbiota at the phylum, class, order, family, genus, and species level in *E. granulosus* s.l. infected mice. **a** The relative abundance of *E. granulosus* s.l. infected and control mice were compared at the level of phylum by Mann Whitney *U* tests (*P* < 0.01); **b** The relative abundance of *E. granulosus* s.l. infected and control mice were compared at the level of class by Mann Whitney *U* tests (*P* < 0.01); **c** The relative abundance of *E. granulosus* s.l. infected and control mice were compared at the level of order by Mann Whitney *U* tests (*P* < 0.01); **d** The relative abundance of *E. granulosus* s.l. infected and control mice were compared at the level of family by Mann Whitney *U* tests (*P* < 0.01); **e** The relative abundance of *E. granulosus* s.l. infected and control mice were compared at the level of genus by Mann Whitney *U* tests (*P* < 0.01); **f** The relative abundance of *E. granulosus* s.l. infected and control mice were compared at the level of species by Mann Whitney *U* tests (*P* < 0.01); **g** from 48 genera with different relative abundances, a co-occurrence network determined that there were significant differences between infected and control mice. According to the species enrichment in the infected and control mice, the species were rearranged on both sides. The negative and positive correlation of the Spearman correlation coefficient are expressed by orange and green edges, respectively. The size of the node indicates the number of genes in each species, and the color of the node indicates the classification at the phylum level (Ctrl: control; Inft: infected)

To determine the specific community associated with infection, the composition of gut microbiota in infected and control mice was compared using Mann–Whitney *U* tests. At the phylum level, nine abundant taxa were identified, the relative abundance of Firmicutes and Proteobacteria were enriched in infected mice, whereas Bacteroidetes and Patescibacteria were enriched in control mice (*P* < 0.01, Fig. 2a). At the class level, 13 abundant taxa were identified, the relative abundance of Bacilli was enriched in control mice, but decreased in infected mice (*P* < 0.01, Fig. 2b). At the order level, 15 abundant taxa were identified, the relative abundance of Lactobacillales was enriched in control mice, but decreased in infected mice (*P* < 0.01, Fig. 2c). At the family level, 28 abundant taxa were identified, enriched bacteria in the infected mice was Streptococcaceae, while the enriched bacteria in the control group was Lactobacillaceae (*P* < 0.01, Fig. 2d). At the genus level, 79 abundant taxa were identified, enriched bacteria in the infected mice was Streptococcus, while the enriched bacteria in the control group was uncultured_bacterium_f_Eggerthellaceae (*P* < 0.01, Fig. 2e). At the species level, 80 abundant taxa were identified, enriched bacteria in the infected mice was uncultured_bacterium_g_Streptococcus, while the enriched bacteria in the control group was uncultured_bacterium_f_Eggerthellaceae (*P* < 0.01, Fig. 2f).

The different genera were used to construct an interactive network to describe the relationship between infected mice and related intestinal microorganisms. In this way, the specific intestinal microorganisms could be identified that can mark the state of infection in mice (*P* < 0.05; Fig. 2g). The differences in gut microbiota identified by the two different methods are almost identical, demonstrating the reliability of the data.

### Metabolic Pathway Analysis of Intestinal Microbiota in E. granulosus s.l. Infected and Control Mice

**Fig. 3:**
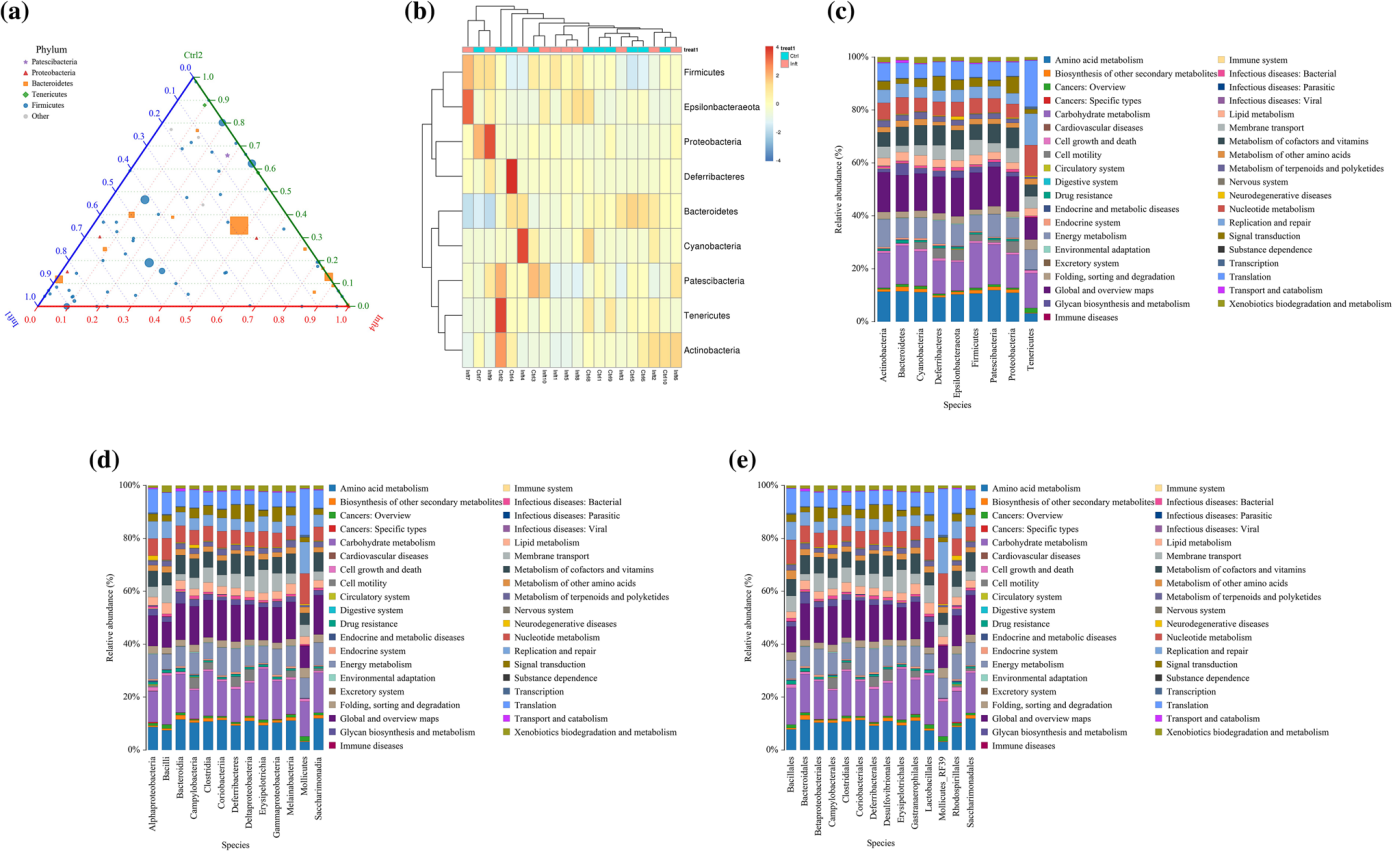

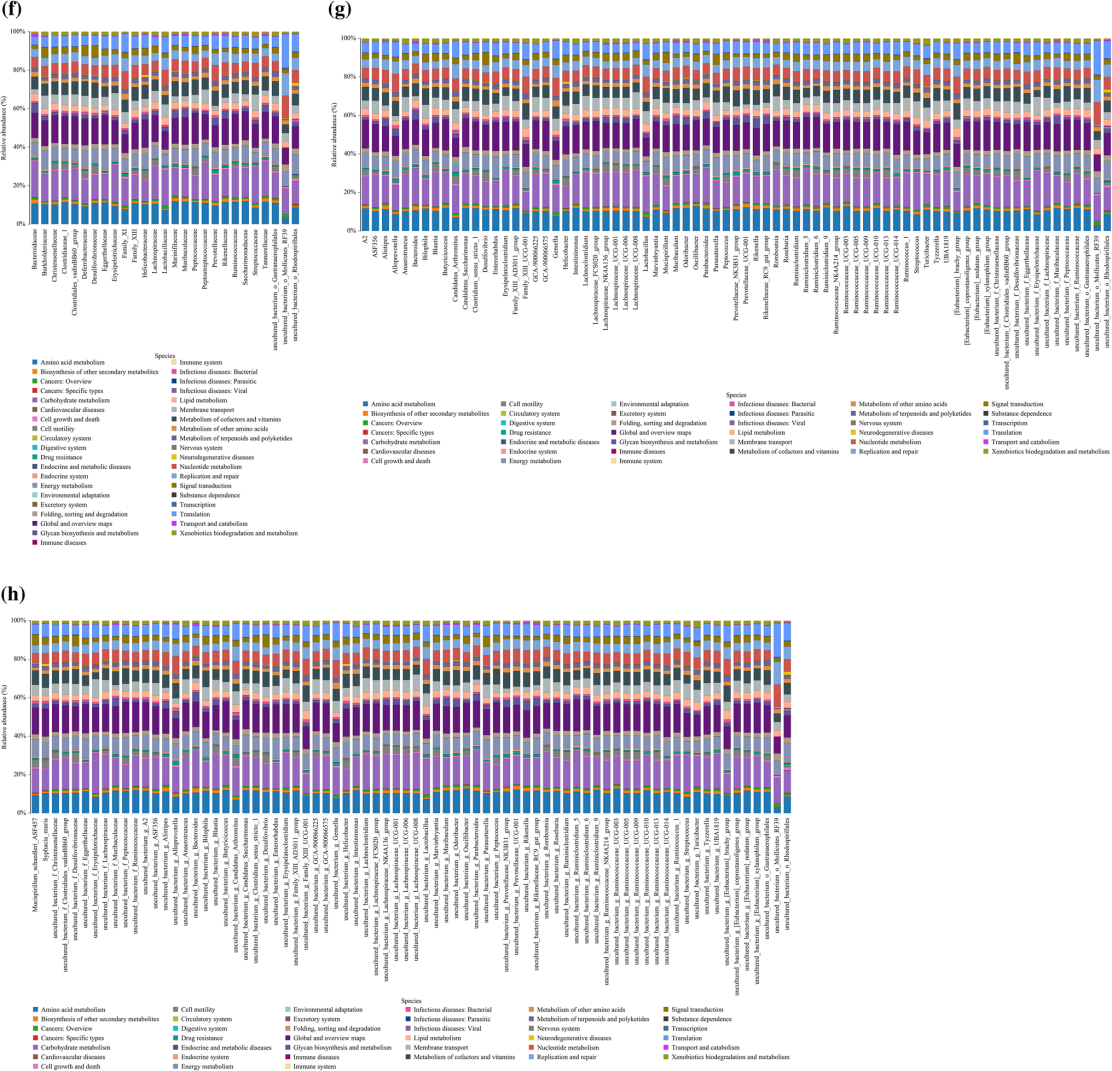
The composition and functional characteristics of intestinal microorganisms in *E. granulosus* s.l. infected and control mice. **a** In the infection model, different microbial populations were selected according to the feature elimination step; **b** heat maps based on the abundance of different microbial populations. Hierarchical clustering (Euclidean distance, complete linkage) showed that the infected mice and the control mice were significantly distinct; The Kyoto Encyclopedia of Genes and Genomes (KEGG) was used to analyze the metabolic pathways involved in microbial populations at different levels of phylum, class, order, family, genus, and species. **c** phylum level, **d** class level, **e** order level, **f** family level, **g** genus level, **h** species level, *Q value < 0.05

From the above results, the intestinal flora with different expression abundance between infected and uninfected mice were screened at different levels. To obtain a more accurate identification of the specific microbial population of mice infected with *E. granulosus* s.l., five phyla were screened out through a feature elimination step (Fig. 3a). At the same time, the microbial population with obvious abundance differences were plotted in all the test samples (10 mice per group) into a heat map. A hierarchical cluster analysis showed that there was a significant separation between the infected and control mice (Fig. 3b). On the other hand, the taxonomic groups of intestinal microbiota were confirmed and provide a good foundation for further study of the role of intestinal flora in the process of echinococcosis infection.

To further analyze the functional changes caused by alterations in the intestinal flora of mice infected with *E. granulosus* s.l., the PICRUSt analysis method was used to predict the functional composition spectrum of intestinal flora in mice infected with *E. granulosus* s.l. More than 200 KEGG pathways were tested, and 39 pathways were identified that were differentially enriched between the infected and control mice (*P* < 0.05) (Fig. 3c–h). The following pathways were closely associated with differential intestinal flora to varying degrees: amino acid metabolism, biosynthesis of other secondary metabolites, carbohydrate metabolism, cell growth and death, lipid metabolism, metabolism of cofactors and vitamins, energy metabolism, nucleotide metabolism, xenobiotics biodegradation and metabolism, metabolism of other amino acids, drug resistance: antimicrobial, endocrine system, signaling molecules and interaction, transport and catabolism, immune system, excretory system, digestive system, substance dependence, infectious diseases: bacterial, and infectious diseases: parasitic, immune diseases.

## Discussion

Cystic echinococcosis has been effectively controlled in some areas (Australia, New Zealand, and Tasmania) in the past few decades [20]. But it is far from achieving the desired effect. Scientific research and clinical research have been looking for more effective methods to treat echinococcosis. In recent years, some researchers believe that the internal relationship between intestinal flora and host is one of the key factors for human and animal health and disease [21]. The host can influence the formation and change of intestinal flora, which can also protect the host from pathogens [22, 23]. Pathogens and other external factors can cause the disorder of intestinal flora of host and induce various diseases [24–27].Therefore, by studying the status and changes of the intestinal flora of the host, we can better understand the diseases of the host.

High-throughput sequencing technology and 16S rRNA sequencing have enabled researchers to achieve rich results in the study of microbial populations [28, 29]. There have been a large number of reports on the effects of parasitic infection on intestinal flora using animals as models [30]. However, there is no report on the effect of *E. granulosus* s.l. infection on the composition of host intestinal flora in mice model. In the study of mice infected with *E. granulosus* s.l., we found that although *E. granulosus* s.l. did not affect the intestinal microbial diversity of mice, it led to the changes of intestinal flora abundance of infected mice at different species levels. Our study found that at the phylum level, the relative abundance of Firmicutes and Proteobacteria in infected mice increased, while the relative abundance of Bacteroidetes decreased. Firmicutes, Proteobacteria, and Bacteroidetes are the dominant flora of all infected and uninfected mice, which is consistent with other research reports on the dominant flora of vertebrates [31]. When *E. granulosus* s.l. infects the host, it will form fibrous vesicles in the body. Firmicutes is the most common intestinal bacteria in the host, and one of its main functions is fiber degradation [32]. The results obtained in this study are consistent with those obtained in other infection models [32]. Proteobacteria is widely distributed in nature. Most of them exist in water, soil, and dirt. They are the normal flora of the host intestine. It is a conditional pathogen that causes infection only when it leaves the normal host intestine and enters other parts of the host. It has been reported that when the host is infected with *Cryptosporidium*, the abundance of Proteobacteria in the body will increase [33]. Our results are consistent with those reported in the literature. Bacteroidetes are probiotics in the gastrointestinal tract of humans and animals. As symbionts, they benefit the host by helping digest complex carbohydrates, biotransformation of bile acids, vitamin synthesis and development of the immune system [34]. This study found that compared with the normal group, mice infected with *E. granulosus* s.l. had a lower abundance of Bacteroidetes, which may be due to the influence of infection on the intestinal digestive dysfunction of mice. It has been reported that reconstructing new intestinal flora can realize the treatment of intestinal and extra-intestinal diseases [35]. First, intestinal microorganisms can resist parasitic infection by affecting the host immune response, for example, induce host immune cells to produce cytokines [36]. In the process of host infection with *E. granulosus* s.l.*,* the host removes pathogens through cellular and humoral immune responses, and the pathogens escape the host immune defense line and parasitize in the host. Previous research of our group demonstrated that the state and proportion of immune cells varied in different stages of *E. granulosus* infection, and the cytokines secreted by immune cells would also increase or decrease [37–39]. In conclusion, different microbial populations in *E. granulosus* s.l. infection screened in this study may affect the progress and prognosis of disease through interaction with the host immune response, as is the case in a number of other diseases [40–42].

To further understand the mechanism of different microbial populations in *E. granulosus* s.l. infection, a KEGG pathway analysis was used to determine the metabolic pathways of microbial populations with different abundances in the infection model. The gut microbiota genera altered by *E. granulosus* s.l. infection were significantly correlated with a number of important pathways, including immune system, infectious diseases: bacterial, infectious diseases: parasitic, and immune diseases. The KEGG prediction results show that the metabolic pathways related to differential microorganisms include parasite infection and immune system diseases. This is consistent with our expectations, and further demonstration of this is currently being undertaken.

Although preliminary results have been achieved in this study, due to limits of the experimental animals and technology, this study has some limitations, therefore, the following suggestions are put forward for future work. First, the changes and effects of intestinal metabolomics and metabolomics on *E. granulosus* s.l. infection were analyzed. Second, since mice are not intermediate hosts of *E. granulosus* s.l., secondary infection is required to produce cysts. Compared with the oral route infection of the intermediate host, the intestinal flora may be affected, so the improvement of the infection method and the selection of the intermediate host as the model animal may achieve more realistic results. Third, a prediction model should be used to detect whether specific intestinal microorganisms can effectively predict the infection status of *E. granulosus* s.l. The follow-up study aims to provide new targets and directions for the treatment of CE, through more in-depth research.
